# Metabolomics reveals the mechanisms of action of fosfomycin and azithromycin combination in the treatment of *Pseudomonas aeruginosa*

**DOI:** 10.3389/fcimb.2025.1663542

**Published:** 2025-10-22

**Authors:** Ziyun Zhao, Shuo Diao, Meng Song, Xiujiao Cao, Yiran Zhao, Mingming Yu, Zhihua Lv, Sherwin K. B. Sy, Hai Yang

**Affiliations:** ^1^ Qingdao Central Hospital, University of Health and Rehabilitation Sciences, Qingdao, China; ^2^ School of Medicine and Pharmacy, Ocean University of China, Qingdao, China; ^3^ Laboratory for Marine Drugs and Bioproducts of Qingdao National Laboratory for Marine Science and Technology, Qingdao, China; ^4^ Department of Statistics, State University of Maringá, Maringá, Paraná, Brazil

**Keywords:** fosfomycin, azithromycin, *Pseudomonas aeruginosa*, metabolomics, combination therapy

## Abstract

**Background:**

Fosfomycin combined with other antibiotics is often used to treat *Pseudomonas aeruginosa* infections. In this study, we investigated the effects of fosfomycin and azithromycin as monotherapy and combination therapy on the metabolic changes of multidrug-resistant *P. aeruginosa*.

**Methods:**

Multidrug-resistant *P. aeruginosa* was exposed to fosfomycin, azithromycin, or their combination. Non-targeted metabolomic profiling was performed using LC–MS/MS. Differential metabolites were identified statistically using Student’s t-test, with significance defined as p < 0.05 and log_2_ fold change (log_2_FC) ≥ 1 or ≤ −1.

**Results:**

The minimum inhibitory concentration was 32/4 μg/mL for fosfomycin/azithromycin combination against the *P. aeruginosa* strain evaluated for metabolomic changes. Metabolomic analysis showed that the combination therapy resulted in greater disturbances affecting the abundance and content levels of metabolites of *P. aeruginosa* than monotherapies. The affected metabolic pathways were mainly amino acid metabolism, nucleotide metabolism, carbohydrate metabolism and lipid metabolism, among which nucleotide metabolism was most significantly disturbed. In the nucleotide metabolism, purine metabolism was affected more than pyrimidine metabolism.

**Conclusion:**

Fosfomycin–azithromycin combination therapy exerted stronger interference on the metabolic pathways of *P. aeruginosa* than either drug alone, indicating more substantial metabolic alterations at the cellular level. These findings provide mechanistic insights that may help inform the potential application of combination regimens against multidrug-resistant *P. aeruginosa* in the clinic.

## 1 Introduction

The increase in prevalence of multidrug-resistant (MDR) bacterial infections constitutes a serious and growing public health concern worldwide (Oo and Sy, 2018; 2020; Oo et al., 2023; Reem et al., 2024). *Pseudomonas aeruginosa* stands out among these pathogens due to its remarkable capacity to adapt to their environment with its intrinsic and acquired resistance mechanisms (Nawaz et al., 2024). This organism commonly causes severe and often life-threatening infections in immunocompromised individuals, including cancer patients, those with severe burns, and people living with cystic fibrosis (Wu et al., 2015). The management of MDR *P. aeruginosa* infections remains particularly difficult, owing to its limited susceptibility to conventional antibiotics. Current therapeutic strategies rely heavily on colistin, which is frequently administered in combination with carbapenems (e.g., imipenem) or aztreonam. However, the utility of colistin is constrained by its dose-dependent nephrotoxicity and increasing prevalence of resistance of pathogens to colistin (Feng et al., 2021). In light of these limitations, recent research has turned toward alternative combination regimens. Fosfomycin, especially when paired with β-lactams or aminoglycosides, has shown encouraging results in enhancing antibacterial efficacy and overcoming resistance, offering a viable therapeutic alternative (Meschiari et al., 2024; Nawaz et al., 2024).

Fosfomycin is a broad-spectrum cell wall synthesis inhibitor for the treatment of uncomplicated cystitis (Sastry and Doi, 2016). Fosfomycin is a promising drug, especially in combination with other drugs, for the treatment of a variety of infections caused by multidrug-resistant Gram-positive and Gram-negative bacteria (Lu et al., 2011). Azithromycin is a semisynthetic macrolide antibiotic known for its favorable tolerance profile and low toxicity (Martinez et al., 2015). It exhibits activity against certain intrinsically resistant pathogens, such as *P. aeruginosa* and *Stenotrophomonas maltophilia*, through mechanisms that include inhibition of bacterial quorum sensing, reduction of biofilm formation, and decreased mucus production (Kumar et al., 2021). Its ability to reduce biofilm formation may also improve the efficacy of other antibiotics (Presterl et al., 2009). Studies have demonstrated that fosfomycin combined with azithromycin has potential applications for *K. pneumoniae* (Gomara-Lomero et al., 2023). In this study, we investigated whether the combination of fosfomycin and azithromycin could exert enhanced inhibitory activity against *P. aeruginosa*. We hypothesized that the two drugs may act synergistically through interconnected metabolic pathways. This study aims to elucidate the potential mechanisms by which the fosfomycin–azithromycin combination inhibits multidrug-resistant *P. aeruginosa* using metabolomic analysis.

## 2 Materials and methods

### 2.1 Antibiotics, reagents, and bacterial isolates

Fosfomycin and azithromycin reference standards were purchased from Shanghai Yuanye Biotechnology Co., Ltd. Following CLSI guidelines (CLSI, 2024), they were dissolved in sterile water and methanol, respectively, to prepare stock solutions at a concentration of 5120 μg/mL and stored at −80°C. The stock solutions were first diluted with Milli-Q water (Millipore, North Rye, Australia) and then passed through 0.22 μm filters to obtain working solutions. Three clinical isolates of *P. aeruginosa* were obtained from the Affiliated Hospital of Qingdao University. The strains were cultured under standard conditions at 35 ± 2°C in a constant-temperature incubator using cation-adjusted Mueller–Hinton broth (CAMHB; Land Bridge, Beijing, China). To ensure the reliability of antimicrobial susceptibility testing, *E. coli* ATCC 25922 and *P. aeruginosa* ATCC 27853 were employed as quality control strains. Next-generation sequencing was performed on the clinical *P. aeruginosa* isolates to determine β-lactamase gene types. Genomic DNA was extracted using the Wizard^®^ Genomic DNA Purification Kit (Promega) according to the manufacturer’s instructions. Sequencing was carried out on an Illumina MiSeq platform. The reads were assembled using SOAPdenovo2, coding sequences were predicted with Glimmer, and the predicted sequences were compared against known drug-resistance genes using BLAST (Zhang et al., 2022, Zhang et al., 2023a).

### 2.2 *In vitro* drug susceptibility testing

The minimum inhibitory concentration (MIC) of fosfomycin and azithromycin, whether applied individually or in combination, were assessed using the broth microdilution technique based on CLSI guidelines (CLSI, 2024). Stock solutions of each antibiotic were serially diluted in Mueller–Hinton broth (MHB) to generate final concentration ranges of 0–512 μg/mL for fosfomycin and 0–128 μg/mL for azithromycin. Bacterial inocula were prepared by adjusting overnight cultures to a turbidity equivalent to 0.5 McFarland standard, followed by a 1:100 dilution in fresh MHB to achieve a final inoculum density of approximately 1 × 10^6^ CFU/mL. Aliquots of 100 μL of drug-containing medium and 100 μL of bacterial suspension were dispensed into sterile 96-well microplates. Plates were incubated at 35 ± 2°C for 20 h under aerobic conditions.

The fractional inhibitory concentration index (FICI) was calculated according to the following equation to evaluate the antimicrobial synergy of the combined use of fosfomycin and azithromycin:


$$\begin{array}{c} \text{FICI}=\frac{\text{MIC of fosfomycin in combination}}{\text{MIC of fosfomycin alone}}+\\ \frac{\text{MIC of azithromycin in combination}}{\text{MIC of azithromycin alone}} \end{array}$$

The interpretive criteria are as follows: FICI ≤0.5, synergistic effect; 0.5<FICI≤1, additive effect; 1<FICI≤2, indifferent effect; FICI>2, antagonistic effect.

### 2.3 Time kill curve study

*P. aeruginosa* strains 6, 12, and 13 were selected to run time-kill studies. Each strain was divided into four groups, namely, blank control group, fosfomycin group, azithromycin group, and fosfomycin/azithromycin combination group. The three strains were inoculated into 15 mL centrifuge tubes containing 10 mL MHB medium and cultured in a 37°C constant temperature incubator (speed of 180 rpm) resulting in a bacterial concentration of 0.5 McFarland. The corresponding antibiotics were added to each bacterial solution so that the final concentration of the antibiotics was the MIC value measured by the *in vitro* drug susceptibility test (Zhou et al., 2024; Ji et al., 2025). The drug concentrations of strains 6, 12, and 13 were: fosfomycin 64, 32, 32 μg/mL; azithromycin 8, 16, 4 μg/mL; and fosfomycin/azithromycin 64/8, 32/16, 32/4 μg/mL, respectively. Each strain was placed in a shaker for further incubation, and its bacterial concentration was measured at 0, 0.25, 0.5, 2, 4, 8 and 24 h.

### 2.4 Extraction of bacterial metabolites

Strain 13 was selected for metabolomic analysis because it had the lowest FICI score for the azithromycin–fosfomycin combination, indicating the strongest synergistic effect, and it also exhibited representative susceptibility and growth characteristics suitable for investigating metabolic responses to drug treatment. The experiment was divided into blank control, fosfomycin, azithromycin and fosfomycin/azithromycin combination groups. A single colony was inoculated into a 15 mL centrifuge tube containing 10 mL MHB and cultured in a 37°C constant temperature incubator (speed of 180 rpm) for 20 hours. The resulting bacterial culture was diluted in MHB to make a bacterial concentration of 0.5 McFarland. Antibiotics were added to a final concentration of 32 μg/mL fosfomycin, 4 μg/mL azithromycin or 32/4 μg/mL fosfomycin/azithromycin.

Bacterial cultures were incubated at 37°C, and aliquots were collected at 0.25, 2, and 4h for metabolite extraction. For each time point, five biological replicates were obtained. A 5 mL volume of bacterial suspension was withdrawn and adjusted to approximately 0.5 McFarland standard using MHB. The samples were transferred into pre-chilled 10 mL centrifuge tubes and centrifuged at 4°C and 3220×g for 10 minutes. The supernatant was discarded, and the bacterial pellets were washed twice with 1 mL of ice-cold normal saline to remove residual media components.

Following the washing step, 500 μL of a pre-cooled extraction solvent containing internal standards was added to each sample. The extraction solution consisted of chloroform, methanol, and water in a 1:1:1 (v/v/v) ratio and included 1 μM of each of the following standards: 3-[(3-cholamidopropyl)-dimethylammonio]-1-propanesulfonate (CHAPS), N-cyclohexyl-3-aminopropanesulfonic acid (CAPS), piperazine-N, N′-bis (2-ethanesulfonic acid) (PIPES), and Tris. The mixtures were immediately frozen in liquid nitrogen, thawed on ice, and vortexed thoroughly to facilitate intracellular metabolite release (Maifiah et al., 2017; Zhu et al., 2023). After metabolite extraction, the samples were centrifuged again at 4 °C and 3220×g for 10 minutes to remove cellular debris. The resulting supernatants (300 μL) were transferred into 1.5 mL Eppendorf tubes and subjected to a second centrifugation at 14,000×g for 10 minutes at 4 °C. A final volume of 200 μL of clear supernatant was transferred into LC-MS/MS sample vials for downstream analysis.

### 2.5 Liquid chromatography-high resolution mass spectrometry

LC-MS conditions were optimized based on previous conditions (Zhu et al., 2022). The samples were analyzed by LC-MS equipped with an Acquity UPLC Ι-Class plus ultrahigh-performance liquid chromatography system and a Synapt XS high-definition mass spectrometer, which operated in both positive and negative (+/-) electrospray ionization (ESI) mode with a detection range of 50–1500 Da. The analytes were separated by a HILIC column (2.1×100 mm, 1.7 μm, HILIC-A, UK) with a column temperature of 40 °C. The mobile phases were an aqueous solution of ammonium formate with a concentration of 10 mM (mobile phase A) and acetonitrile (mobile phase B). The elution conditions were as follows: 0 to 0.5 min 5% A phase, 0.5 to 8 min transition from 5% A phase to 25% A phase, 8 to 10 min transition from 25% A phase to 60% A phase, 10 to 12 min isocratic elution at 60% A phase, 12 to 12.5 min transition from 60% A phase to 5% A phase, 12.5 to 15 min isocratic elution at 5% A phase. The injection volume was 10 μL. The flow rate was 0.3 mL/min.

### 2.6 Data analysis

Raw LC-MS data were imported into Progenesis QI software (Waters, USA) for preprocessing, including retention time correction, feature detection, and intensity normalization. Compound identification was achieved by matching retention characteristics and accurate mass-to-charge (m/z) values.

The processed data matrix was subsequently uploaded to the MetaboAnalyst 5.0 platform (https://www.metaboanalyst.ca/) for multivariate and statistical analysis. Principal component analysis (PCA) was applied to visualize the clustering patterns among different experimental groups. For univariate analysis, one-way ANOVA was initially performed to assess global metabolic variation, followed by Student’s t-test to detect significant pairwise differences. Features were considered differentially expressed if they met the criteria of p < 0.05 (adjusted for false discovery rate, FDR) and exhibited a fold change (FC) ≥ 2, corresponding to log_2_FC ≥ 1 or ≤ −1. To assist with metabolite annotation and biological interpretation, pathway enrichment analysis and compound classification were conducted using the Kyoto Encyclopedia of Genes and Genomes (KEGG) and the Human Metabolome Database (HMDB) (Zhang et al., 2023b).

## 3 Results

### 3.1 *In vitro* drug susceptibility testing

The *in vitro* susceptibility profiles of the tested strains are summarized in **Table 1**. According to CLSI breakpoints (fosfomycin: susceptible, ≤64 μg/mL; intermediate, 128 μg/mL; resistant, ≥256 μg/mL; azithromycin: susceptible, ≤16 μg/mL; resistant, ≥32 μg/mL), the reference strains *E. coli* ATCC 25922 and *P. aeruginosa* ATCC 27853 were susceptible to both fosfomycin and azithromycin, with MIC values of 16 and 4 μg/mL, respectively. In contrast, all three clinical *P. aeruginosa* isolates harbored OXA-type resistance genes and exhibited resistance to either fosfomycin (MIC, 128–512 μg/mL) or azithromycin (MIC, 32–64 μg/mL) when tested alone. Notably, the combination of fosfomycin and azithromycin markedly decreased the MIC values to the susceptibility breakpoint or below, with fosfomycin MIC reduced to 32–64 μg/mL and azithromycin MIC reduced to 4–16 μg/mL. Synergistic effects (FICI ≤ 0.5) were observed in two of the three isolates, with the strongest synergy observed in strain 13 (FICI = 0.1875). These findings highlight the potential of fosfomycin–azithromycin combination therapy to overcome resistance in clinical *P. aeruginosa* isolates.

**Table 1:** Minimum inhibitory concentration (MIC) of fosfomycin alone, azithromycin alone and fosfomycin/azithromycin combination against *P. aeruginosa*, as well as drug resistance genes encoded in each isolate.

Strains	Drug resistance genes encoded	MIC (μg/mL)			FICI
		Fosfomycin	Azithromycin	Fosfomycin/Azithromycin	
*E. coli* ATCC 25922		16	4	–	–
*P. aeruginosa* ATCC 27853		16	4	–	–
Pae strain 6^a^	OXA-396, OXA-101, OXA-494, PAO, PER-1	512	64	64/8	0.2500
Pae strain 12^b^	OXA-50, PAO	128	64	32/16	0.5000
Pae strain 13^c^	OXA-488, PAO	512	32	32/4	0.1875

MIC, minimum inhibitory concentration; FICI, fractional inhibitory concentration index; CLSI breakpoints for interpretation of fosfomycin MIC results: ≤64 μg/mL (susceptible), 128 μg/mL (intermediate), and ≥256 μg/mL (resistant); and azithromycin MIC results: ≤16 μg/mL (susceptible) and ≥32 μg/mL (resistant) for *P. aeruginosa*.

a P. aeruginosa strain 6.

b *P. aeruginosa* strain 12.

c *P. aeruginosa* strain 13.

### 3.2 Combined fosfomycin/azithromycin effect on metabolomic changes in *Pseudomonas aeruginosa*

**Figure 1:**
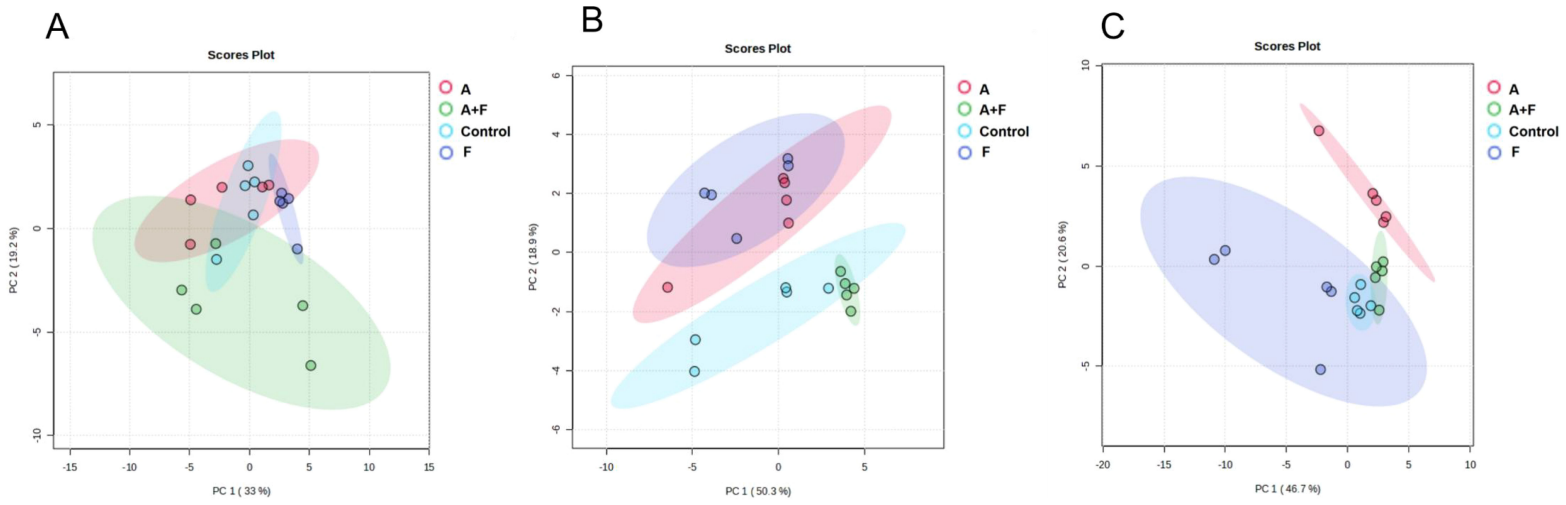
Principal component analysis (PCA) plots of metabolite levels of *P. aeruginosa* in the control group (Control), fosfomycin group (F), azithromycin group (A) and fosfomycin/azithromycin combination group (A+F) at 15 min **(A)**, 2 h **(B)** and 4 h **(C)**.

**Figure 2:**
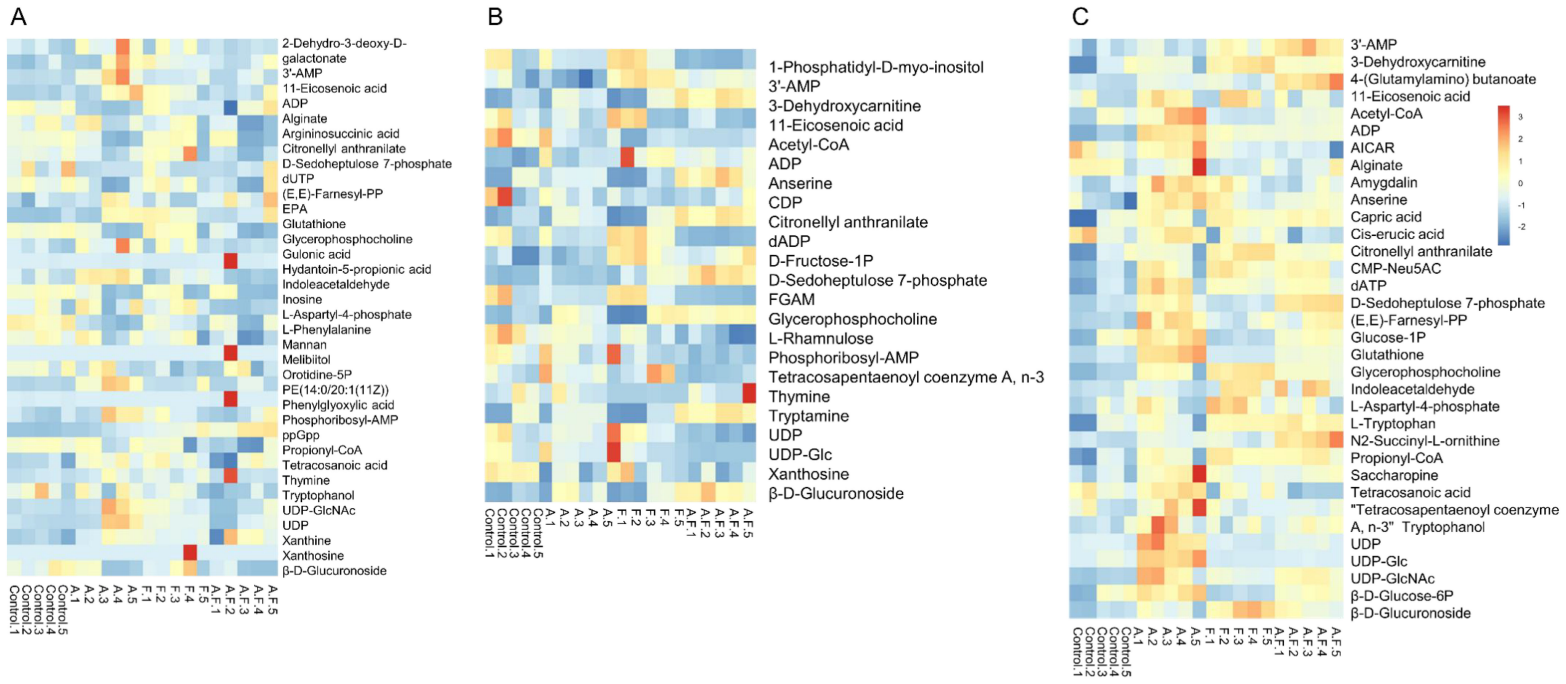
Heatmap of fosfomycin group (F), azithromycin group (A) alone and fosfomycin/azithromycin combination (A.F) against *P. aeruginosa* strains at 15 min **(A)**, 2 h **(B)** and 4 h **(C)**, Numbers 1–5 indicate five biological replicates for each group.

A total of 59 metabolites affected by fosfomycin and azithromycin were identified via KEGG and HMDB databases. PCA (**Figure 1**) and heatmap (**Figure 2**) analyses indicated clear differences between the combination therapy group and the monotherapy or control groups, particularly at early time points (15 min and 2 h). The affected metabolites were grouped into amino acid, nucleotide, carbohydrate, and lipid metabolism (**Supplementary Table S1**, **Supplementary Figure S1**).

### 3.3 Effects of fosfomycin and azithromycin alone or in combination on amino acid metabolism of *Pseudomonas aeruginosa*

**Figure 3:**
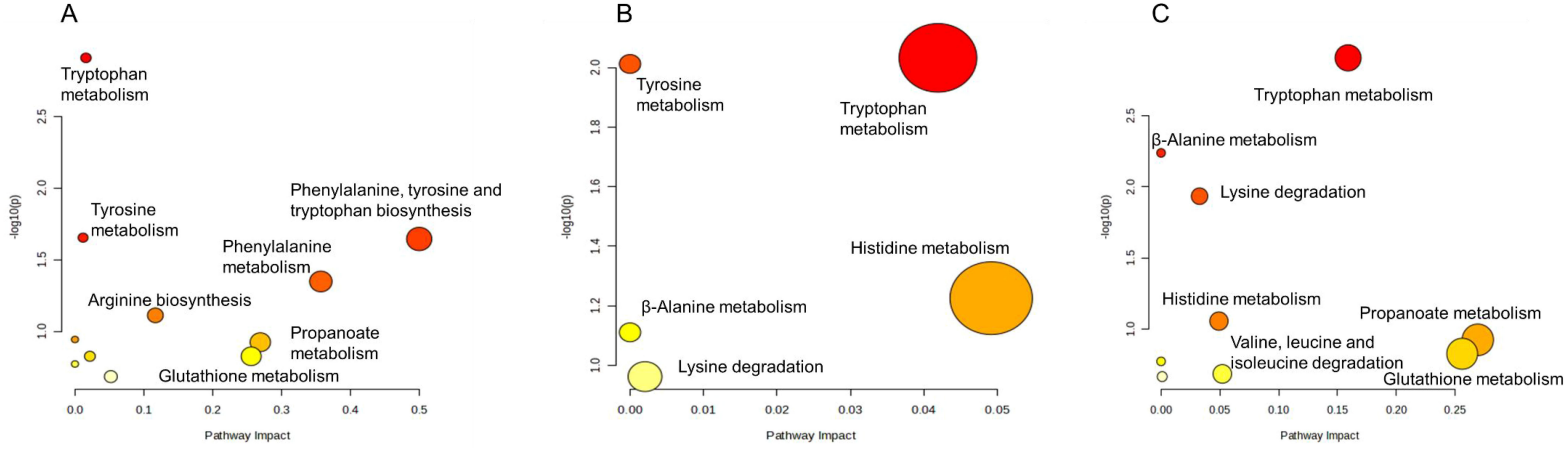
Enrichment bubble plots of fosfomycin and azithromycin alone and in combination showing the disruption of amino acid metabolism in *P. aeruginosa* at 15 min **(A)**, 2 h **(B)** and 4 h **(C)**. Significantly perturbed metabolites were selected according to Log_2_FC ≤−1 or ≥1, p <0.05.

The combination of fosfomycin and azithromycin affected the levels of 18 amino acid metabolites at 15 min, 2 and 4 h. At 15 min, six amino acids in the combination therapy group were decreased, including L-phenylalanine, L-aspartyl-4-phosphate, argininosuccinic acid, tryptophanol, indoleacetaldehyde, and propionyl-CoA (Log_2_FC = −1.83 to −1.00), while two amino acids were upregulated. At 2 h, tryptophanol (Log_2_FC = 1.13 to 1.51) and 3-dehydroxycarnitine (Log_2_FC = 1.02 to 1.64) levels in the combination therapy group were increased. At 4 h, all affected amino acids in the combination therapy group were elevated. These results suggest that the combination therapy may influence amino acid metabolism pathways by modulating the levels of key amino acid metabolites (**Figure 3**).

### 3.4 Effects of fosfomycin and azithromycin alone or in combination on nucleotide metabolism of *Pseudomonas aeruginosa*

**Figure 4:**
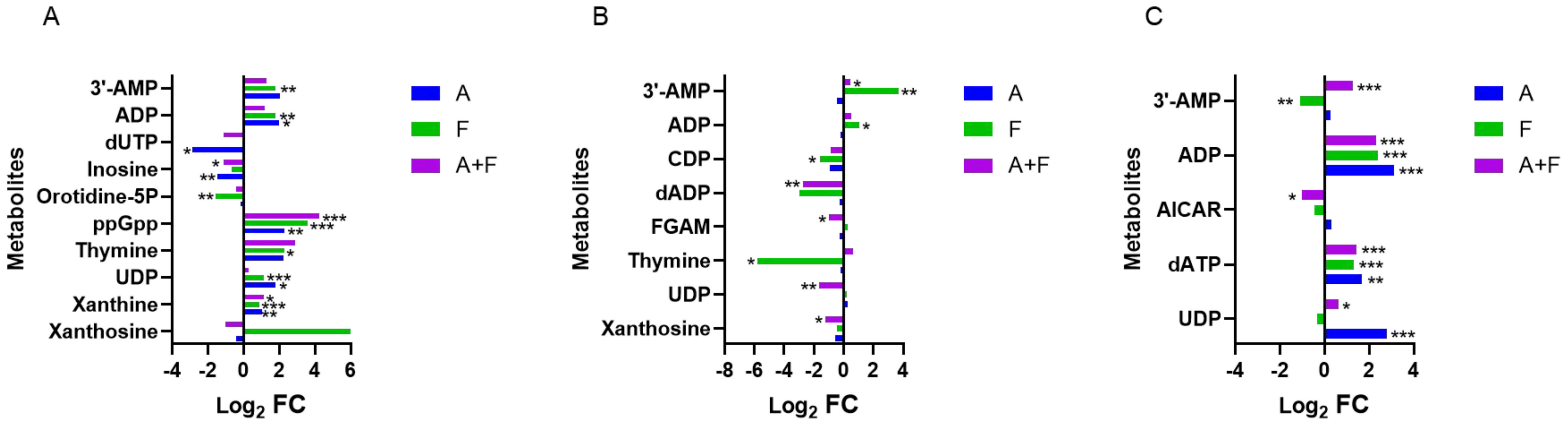
Interference of the nucleotide metabolism pathway of *P. aeruginosa* at 15 min **(A)**, 2 h **(B)** and 4 h **(C)**, by fosfomycin and azithromycin alone and in combination. Significantly perturbed metabolites were selected based on Log_2_FC ≤−1 or ≥1, p <0.05; *p <0.05; **p <0.01; ***p <0.001.

The combination of fosfomycin and azithromycin caused disturbances in 15 nucleotides. Within purine metabolism, marked alterations were observed: at 15 min, four nucleotides were upregulated (Log_2_FC = 1.13 to 4.23), with guanosine 3′,5′-bisdiphosphate (ppGpp) showing a significant increase (Log_2_FC = 4.23); at 2 h, FGAM, dADP, and xanthosine were downregulated (Log_2_FC = −2.74 to −1.01); and at 4 h, dATP, ADP, and 3′-AMP were upregulated (Log_2_FC = 1.28 to 3.10), whereas AICAR was slightly downregulated (Log_2_FC = −1.00). Compared with these pronounced changes in purine metabolism, alterations in pyrimidine metabolism were relatively limited, involving the early downregulation of orotidine-5P and dUTP at 15 min (Log_2_FC = −2.85, −1.11) and subsequent shifts in UDP, thymine, and CDP at 2 to 4 h. These findings suggest that the combination therapy may modulate nucleotide metabolism pathways primarily by affecting key purine-related nucleotides such as ppGpp, ADP, dADP, dATP, xanthosine, and 3′-AMP (**Figure 4**).

### 3.5 Effects of fosfomycin and azithromycin alone or in combination on amino sugar and nucleotide sugar metabolism, peptidoglycan biosynthesis and other carbohydrate metabolism in *Pseudomonas aeruginosa*

**Figure 5:**
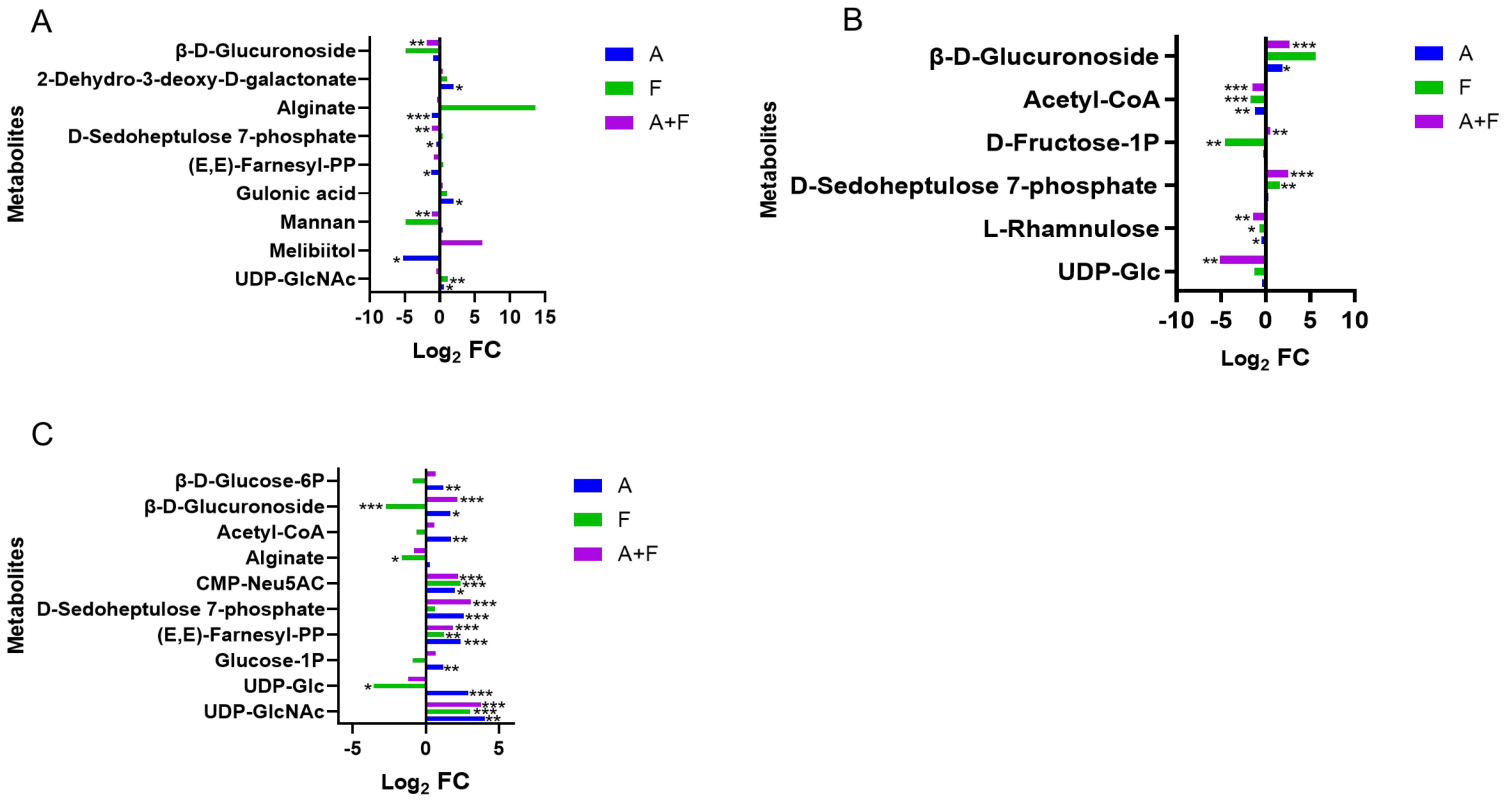
Interference of the carbohydrate metabolism pathway of *P. aeruginosa* at 15 min **(A)**, 2 h **(B)** and 4 h **(C)** by fosfomycin and azithromycin alone and in combination. Significantly perturbed metabolites were selected according to Log_2_FC ≤−1 or ≥1, p <0.05; *p <0.05; **p <0.01; ***p <0.001.

The combination of fosfomycin and azithromycin had limited effects on amino sugar, nucleotide sugar metabolism, and peptidoglycan biosynthesis within the first 2 hours, but at 4 h, it upregulated CMP-Neu5AC (Log_2_FC = 2.18), (E,E)-farnesyl-PP (Log_2_FC = 1.82), and UDP-GlcNAc (Log_2_FC = 3.77), while downregulating UDP-Glc (Log_2_FC = −1.24). In the pentose phosphate pathway, D-sedoheptulose 7-phosphate was downregulated at 15 min (Log_2_FC = −1.08) and upregulated at 2 and 4 h (Log_2_FC = 2.54, 3.09). β-D-glucuronoside was downregulated at 15 min (Log_2_FC = −1.82) and upregulated at 4 h (Log_2_FC = 2.14) by the combination therapy. Central carbon metabolism was largely unchanged, with only minor fluctuations in acetyl-CoA, glucose-1P, and β-D-glucose-6P at 2 to 4 h. These results suggest that the combination therapy may subtly influence carbohydrate metabolism pathways through key intermediates such as CMP-Neu5AC, UDP-GlcNAc, D-sedoheptulose 7-phosphate, and β-D-glucuronoside (**Figure 5**).

### 3.6 Effects of fosfomycin and azithromycin alone or in combination on lipid metabolism of *Pseudomonas aeruginosa*

**Figure 6:**
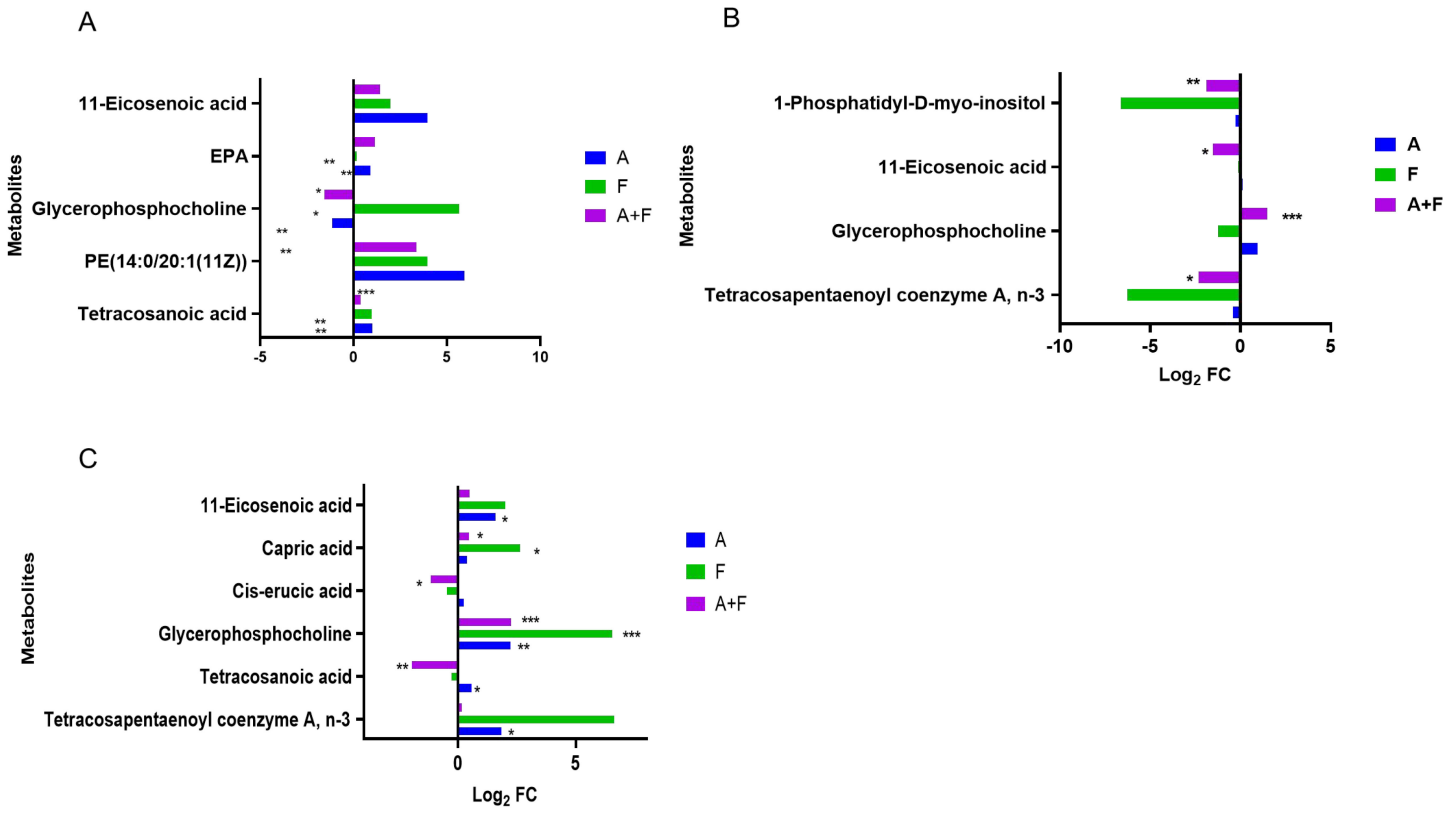
Interference of the lipid metabolism pathway of *P. aeruginosa* at 15 min **(A)**, 2 h **(B)** and 4 h **(C)** by fosfomycin and azithromycin alone and in combination. Significantly perturbed metabolites were selected according to Log_2_FC ≤−1 or ≥1, p <0.05; *p <0.05; **p <0.01; ***p <0.001.

Fosfomycin and azithromycin alone or in combination affected lipid metabolism, including unsaturated fatty acid biosynthesis, glycerophospholipid metabolism, and fatty acid biosynthesis. The combination therapy upregulated 11-eicosenoic acid and eicosapentaenoic acid (EPA) at 15 min (Log_2_FC = 1.42–3.95 and 1.15, respectively) and downregulated 11-eicosenoic acid, tetracosanoic acid, and cis-erucic acid at later time points (Log_2_FC = −1.52 to −1.17). Glycerophosphocholine was transiently downregulated at 15 min (Log_2_FC = −1.56) and subsequently upregulated at 2 h and maintained for over 4 hours (Log_2_FC = 1.49–2.26). These results indicate that the combination therapy may modulate lipid metabolism pathways by altering the levels of key lipids such as 11-eicosenoic acid, EPA, tetracosanoic acid, cis-erucic acid, and glycerophosphocholine. (**Figure 6**).

## 4 Discussion

Multidrug-resistant *P. aeruginosa* poses a significant threat to public health, particularly in immunocompromised individuals (Breidenstein et al., 2011; Poole, 2011; Oliver et al., 2015). Given the scarcity of newly developed antibiotics, fosfomycin has attracted renewed attention due to its unexplored potential (Raz, 2012; Karaiskos and Giamarellou, 2014). Previous studies have reported that fosfomycin shows synergistic effects when combined with carbapenems (Albiero et al., 2016, Albiero et al., 2019; Horcajada et al., 2019). We demonstrate that fosfomycin also exhibits a synergistic effect with the macrolide antibiotic azithromycin. In this study, metabolomics was applied to explore the potential mechanisms underlying the synergistic effect of fosfomycin and azithromycin against *P. aeruginosa*. The combination therapy disrupted multiple metabolic pathways, including amino acid, nucleotide, carbohydrate, and lipid metabolism.

Azithromycin has previously been reported to disrupt *P. aeruginosa* biofilm formation (Hansen et al., 2005). After combined treatment with fosfomycin and azithromycin, several metabolites related to biofilm formation in *P. aeruginosa* were altered. Glycerophosphocholine and cis-erucic acid, which belong to glycerophospholipids and unsaturated fatty acids, respectively, are known to contribute to biofilm structure (Bleijerveld et al., 2006; Vollhardt, 2007). UDP-Glc participates in the biosynthesis of capsular polysaccharides, lipopolysaccharides, and other membrane-derived oligosaccharides (Zou et al., 2013). The observed changes in these metabolites suggest a potential influence of the combination therapy on biofilm-related pathways, which may partially contribute to the inhibition of bacterial growth. In addition, D-sedoheptulose 7-phosphate, a key metabolite of the pentose phosphate pathway (Charmantray et al., 2009) is required for the biosynthesis of ADP-L-β-D-heptose, a lipopolysaccharide precursor (Kneidinger et al., 2002). Its downregulation under drug exposure may transiently inhibit biofilm formation. Fosfomycin is an old antibiotic that kills bacteria by inhibiting peptidoglycan synthesis, thereby increasing cell permeability (Falagas et al., 2016; Fedrigo et al., 2017). The disruption of membrane stability likely facilitated greater intracellular drug penetration, amplifying the antibacterial effect of the companion drug.

The combination therapy exerted a particularly strong effect on nucleotide metabolism, an essential pathway for bacterial growth and replication (Lopatkin and Yang, 2021). Compared with monotherapy, the combined use of fosfomycin and azithromycin caused more pronounced disturbances. The increase in ADP content may provide feedback inhibition on the rate-limiting step of purine biosynthesis (Chandel, 2021). Concurrently, the decrease in FGAM and AICAR levels could limit the synthesis of inosinic acid, thereby potentially reducing the availability of purine nucleotides for DNA and RNA synthesis. The observed increase in xanthine suggests accelerated purine degradation (Zrenner and Ashihara, 2011). Taken together, these metabolic changes indicate that the combination therapy may impair nucleotide availability, thereby contributing to the synergistic antibacterial effect through interference with bacterial genetic material synthesis and replication. Perturbations in purine metabolism have been shown to influence *P. aeruginosa’s* antibiotic susceptibility and virulence phenotypes, including biofilm formation and quorum-sensing behaviors. For instance, exogenous purines such as adenosine and inosine can reduce intracellular c-di-GMP levels and suppress biofilm development in *P. aeruginosa* (Kennelly et al., 2024). These findings suggest that the metabolic changes observed in this study are consistent with previously reported roles of purine metabolism in regulating bacterial physiology and drug response.

The mechanism of action of azithromycin is to cause cell death by inhibiting protein synthesis, which is consistent with the results of this study; the metabolic levels of a large number of amino acids and peptides were affected. Acetyl-CoA is a product of glucose, fatty acid and amino acid metabolism (Shi and Tu, 2015). The downregulation of acetyl-CoA metabolic levels is due to the effect of fosfomycin/azithromycin combination causing varying degrees of disturbance in multiple metabolic pathways. UDP-GlcNAc and (E, E)-farnesyl-PP were upregulated to varying degrees in the three groups. Both are precursors of UDP-MurNAc (Barreteau et al., 2008). It was suggested that fosfomycin and azithromycin might inhibit the related enzymes that synthesize UDP-MurNAc, causing the levels of the two metabolites to increase, thereby interfering with the formation of bacterial cell walls.

This study has several limitations. Only a single *P. aeruginosa* strain was tested, and all experiments were conducted *in vitro*, which may not fully reflect clinical diversity or *in vivo* conditions. In addition, no randomized controlled trials have assessed the clinical relevance of this combination therapy.

In summary, this metabolomics study demonstrated that fosfomycin combined with azithromycin caused more pronounced metabolic disturbances in *P. aeruginosa* than either drug alone. The combination therapy altered multiple key metabolic pathways, including nucleotide, amino acid, carbohydrate, and lipid metabolism, leading to an impaired biofilm formation and disruption of essential biosynthetic processes. These findings provide mechanistic insights into the synergistic antibacterial effect of fosfomycin and azithromycin and highlight the potential of repurposing this combination as an effective strategy against multidrug-resistant *P. aeruginosa.*

## Data Availability

The original contributions presented in the study are included in the article/**Supplementary Material**. Further inquiries can be directed to the corresponding authors.
