# Supplementary material for: Metabolomics reveals the mechanisms of action of fosfomycin and azithromycin combination in the treatment of Pseudomonas aeruginosa
Source: Front Cell Infect Microbiol. 2025 Oct 22;15:1663542. doi: 10.3389/fcimb.2025.1663542 (PMC12585984; doi:10.3389/fcimb.2025.1663542)
Supplement: Supplementary file 1 [file Table1.docx]

**Table S1: Sequence of metabolomic changes in *P. aeruginosa* following treatment of fosfomycin and azithromycin as monotherapy and combination therapy.**

|  | **Fosfomycin** | **Azithromycin** | **Fosfomycin/Azithromycin** |
| --- | --- | --- | --- |
| **15 min** | **Carbohydrate metabolism**  UDP-GlcNAc ↑  Gulonic acid ↑  ADP ↓  2-Dehydro-3-deoxy-D-galactonate ↑  Alginate ↑  Mannan ↓ | **Carbohydrate metabolism**  (E, E)-Farnesyl-PP ↓  Gulonic acid ↑  2-Dehydro-3-deoxy-D-galactonate ↑  Melibiitol ↓  Alginate ↓ | **Carbohydrate metabolism**  β-D-Glucuronoside ↓  Mannan ↓  Melibiitol ↑  D-Sedoheptulose 7-phosphate ↓ |
|  | **Lipid metabolism**  11-Eicosenoic acid ↑  PE (14:0/20:1(11Z)) ↑  Glycerophosphocholine ↑ | **Lipid metabolism**  Tetracosanoic acid ↑  11-Eicosenoic acid ↑  PE (14:0/20:1(11Z)) ↑  Glycerophosphocholine ↓ | **Lipid metabolism**  EPA ↑  11-Eicosenoic acid ↑  PE (14:0/20:1(11Z)) ↑  Glycerophosphocholine ↓ |
|  | **Nucleotide metabolism**  UDP ↑  3'-AMP ↑  ppGpp ↑  ADP ↑  Orotidine-5P ↓  Thymine ↑  Xanthosine ↑ | **Nucleotide metabolism**  UDP ↑  3'-AMP ↑  ppGpp ↑  Xanthine ↑  ADP ↑  dUTP ↓  Inosine ↓  Thymine ↑ | **Nucleotide metabolism**  3'-AMP ↑  ADP ↑  dUTP ↓  ppGpp ↑  Xanthine ↑  Inosine ↓  Thymine ↑ |
|  | **Amino acid metabolism**  Glutathione ↑  Tryptophanol↓  Argininosuccinic acid↑  Citronellyl anthranilate↑  Hydantoin-5-propionic acid ↓  Phosphoribosyl-AMP ↑ | **Amino acid metabolism**  L-Phenylalanine ↓  Phenylglyoxylic acid ↑  Citronellyl anthranilate ↓  Indoleacetaldehyde ↑  Phosphoribosyl-AMP ↑  Hydantoin-5-propionic acid ↓ | **Amino acid metabolism**  L-Phenylalanine ↓  Phenylglyoxylic acid↑  L-Aspartyl-4-phosphate ↓  Argininosuccinic acid ↓  Tryptophanol ↓  Indoleacetaldehyde ↓  Propionyl-CoA ↓  Hydantoin-5-propionic acid↑ |
| **2 h** | **Carbohydrate metabolism**  UDP-Glc ↓  Acetyl-CoA ↓  D-Fructose-1P ↓  D-Sedoheptulose 7-phosphate ↑  β-D-Glucuronoside ↑ | **Carbohydrate metabolism**  Acetyl-CoA ↓  β-D-Glucuronoside ↑ | **Carbohydrate metabolism**  UDP-Glc ↓  Acetyl-CoA ↓  L-Rhamnulose ↓  D-Sedoheptulose 7-phosphate ↑  β-D-Glucuronoside ↑ |
|  | **Lipid metabolism**  1-Phosphatidyl-D-myo-inositol ↓  Glycerophosphocholine ↓  Tetracosapentaenoyl coenzyme A, n-3 ↓ | **Lipid metabolism** | **Lipid metabolism**  1-Phosphatidyl-D-myo-inositol ↓  Glycerophosphocholine ↑  Tetracosapentaenoyl coenzyme A, n-3 ↓  11-Eicosenoic acid ↓ |
|  | **Nucleotide metabolism**  3'-AMP ↑  dADP ↓  Thymine ↓  CDP ↓  ADP ↑ | **Nucleotide metabolism** | **Nucleotide metabolism**  UDP ↓  FGAM ↓  dADP ↓  Xanthosine ↓ |
|  | **Amino acid metabolism**  Phosphoribosyl-AMP ↓  Citronellyl anthranilate ↓  Tryptamine ↑  3-Dehydroxycarnitine ↑ | **Amino acid metabolism** | **Amino acid metabolism**  Citronellyl anthranilate ↑  Tryptamine ↑  3-Dehydroxycarnitine ↑  Phosphoribosyl-AMP ↓  Anserine ↑ |
| **4 h** | **Carbohydrate metabolism**  UDP-Glc ↓  CMP-Neu5AC ↑  UDP-GlcNAc ↑  (E, E)-Farnesyl-PP ↑  β-D-Glucuronoside ↓  Alginate ↓ | **Carbohydrate metabolism**  UDP-Glc ↑  CMP-Neu5AC ↑  UDP-GlcNAc ↑  (E, E)-Farnesyl-PP ↑  β-D-Glucuronoside ↑  D-Sedoheptulose 7-phosphate ↑  Glucose-1P ↑  Β-D-Glucose-6P ↑  Acetyl-CoA ↑ | **Carbohydrate metabolism**  CMP-Neu5AC ↑  UDP-GlcNAc ↑  (E, E)-Farnesyl-PP ↑  UDP-Glc ↓  β-D-Glucuronoside ↑  D-Sedoheptulose 7-phosphate ↑ |
|  | **Lipid metabolism**  Tetracosapentaenoyl coenzyme A, n-3 ↑  Glycerophosphocholine ↑  Capric acid ↑  11-Eicosenoic acid ↑ | **Lipid metabolism**  Glycerophosphocholine ↑  Tetracosapentaenoyl coenzyme A, n-3 ↑  11-Eicosenoic acid ↑ | **Lipid metabolism**  Glycerophosphocholine ↑  Tetracosanoic acid ↓  Cis-erucic acid ↓ |
|  | **Nucleotide metabolism**  3'-AMP ↓  dATP ↑  ADP ↑ | **Nucleotide metabolism**  UDP ↑  dATP ↑  ADP ↑ | **Nucleotide metabolism**  3'-AMP ↑  dATP ↑  AICAR ↓  ADP ↑ |
|  | **Amino acid metabolism**  L-Aspartyl-4-phosphate ↓  4-(Glutamylamino) butanoate ↓  Tryptophanol ↓  Saccharopine ↑  Citronellyl anthranilate ↑  N_2_-Succinyl-L-ornithine ↑  L-Tryptophan ↑  Propionyl-CoA ↑ | **Amino acid metabolism**  4-(Glutamylamino) butanoate ↑  Tryptophanol ↑  N_2_-Succinyl-L-ornithine ↑  Propionyl-CoA ↑  Glutathione ↑  Anserine ↑ | **Amino acid metabolism**  4-(Glutamylamino) butanoate ↑  Tryptophanol ↑  N_2_-Succinyl-L-ornithine ↑  3-Dehydroxycarnitine ↑  Indoleacetaldehyde ↑  L-Tryptophan ↑  Propionyl-CoA ↑  Glutathione ↑ |


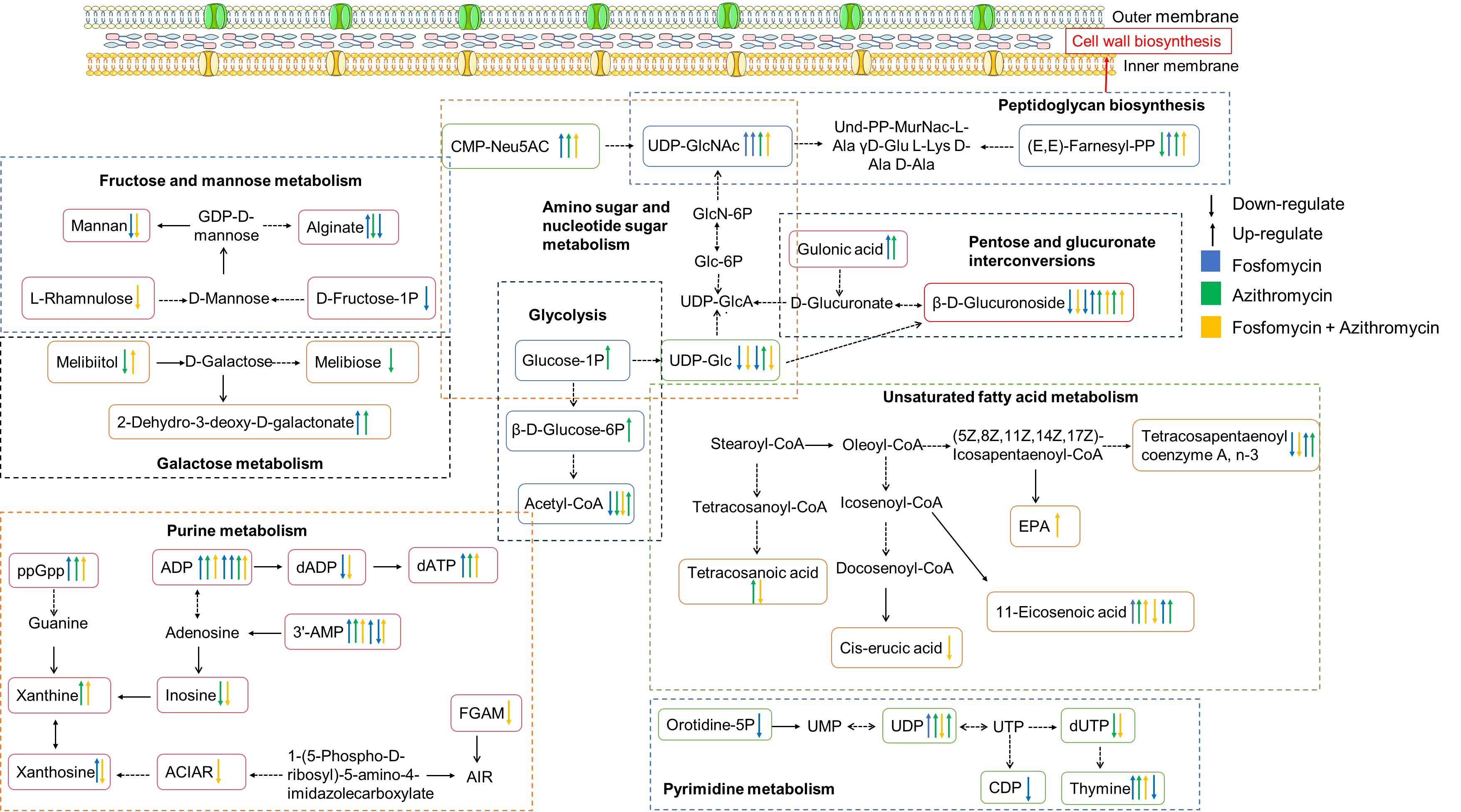


Figure S1: Diagram of metabolic pathways affected by fosfomycin and azithromycin as monotherapy and combination therapy against *P. aeruginosa*.
